# Potential SARS-CoV-2 Preimmune IgM Epitopes

**DOI:** 10.3389/fimmu.2020.00932

**Published:** 2020-04-30

**Authors:** Velizar Shivarov, Peter K. Petrov, Anastas D. Pashov

**Affiliations:** ^1^Laboratory of Clinical Immunology and Department of Clinical Hematology, Sofiamed University Hospital, Sofia, Bulgaria; ^2^Faculty of Biology, Sofia University, Sofia, Bulgaria; ^3^Department of Analysis, Geometry and Topology, Institute of Mathematics and Informatics, Bulgarian Academy of Sciences (BAS), Sofia, Bulgaria; ^4^Department of Immunology, Stephan Angeloff Institute of Microbiology, Bulgarian Academy of Sciences (BAS), Sofia, Bulgaria

**Keywords:** SARS-CoV-2, epitope, mimotope, B cell precursors, IgM

## Abstract

While studying the human public IgM igome as represented by a library of 224,087 linear mimotopes, three exact matches to peptides in the proteins of SARS-CoV-2 were found: two in the open reading frame 1ab and one in the spike protein. Joining the efforts to fast track SARS-CoV-2 vaccine development, here we describe briefly these potential epitopes in comparison to mimotopes representing peptides of SARS-CoV, HCoV 229E and OC43.

## Introduction

The COVID19 pandemic has put to test the capacity of vaccinology to produce as fast as possible relevant vaccines. A number of recent reports predict possible SARS-CoV-2 epitopes for vaccine development but there are no reports on experimentally defined B cell epitopes (1–5). The closest to identification of actual epitopes is the finding of pentapeptide sequences from the viral proteome in other known epitopes form IEDB (5). A library of 224,087 mimotopes corresponding to the human public IgM repertoire as represented in a plasma pool from 10,000 healthy donors was recently designed (6). The mimotopes were selected from a commercial 7 amino acid random peptide phage display library (Ph.D. 7, New England Biolabs). Conceptually, this mimotope library reflects at a certain level of detail, the repertoire of IgM specificities in the plasma focusing on the recurring ones. The latter can be just natural antibodies or they may represent the product of fast extrafollicularly expanding IgM clones that may serve as precursors of highly specific, somatically mutated, class-switched B cells. The preimmune repertoire has to be quasi-complete to provide for rapid expansion of clones reactive with any newly encountered antigen. The same may not be true for our library although, due to the polyspecific binding, most of the available public repertoire may be partially represented in it (6). Here we report that the IgM mimotope library contains heptapeptides identical to peptides in the proteome of SARS-CoV-2. One of them may serve as a potentially neutralizing epitope on the spike protein.

## Methods

The design and the properties of the mimotope database has been published elsewhere (6). The available sequences of the genomes of SARS-CoV (NC_004718.3), SARS-CoV-2 (ASM985889v3), HCoV229E (NC_002645.1), and HCoVOC43 (AY391777.1) were split into consecutive overlapping heptamers shifted by one residue and the resultant sequence sets were compared to the sequences in the database of natural mimotopes. Only exact matches were considered.

The homologous sequences in the non-redundant databases of the human proteome and Viridae (taxid:10239) were blast searched using the NCBI blastp suite (https://blast.ncbi.nlm.nih.gov/Blast.cgi?PAGE=Proteins).

As part of an ongoing analysis, the natural mimotope database was represented as a graph by connecting the sequences having at least 5 exact matches (i.e., of maximal Hamming distance 2). The graph had one giant component containing approximately 90% of the sequences which was further considered as the graph of interest. For the present study, the degrees of the vertices representing the natural SARS-CoV-2 epitopes, all of which belonged to the giant component, were used as the number of adjacent mimotopes parameter. For a set of words of length l based on an alphabet of L symbols, the theoretical average number of neighbors N at Hamming distance D was calculated using the following formula for the number of neighbors:

$$\begin{array}{l} N\left(D,l\right)={\left(L-1\right)}^{D}.\left(\begin{array}{c} l\\ D \end{array}\right) \end{array}$$

For the present study, *L* = 20, *l* = 7, and *D* < 3. For the first layer of neighbors N1 = 133 and for the second N2 = 7581. Under the hypothesis that the database is a random sample from the set of heptamer peptides, the probability of the occurrence of each neighbor is:

$$\begin{array}{l} \text{p}=\;224087/20^{7}\approx 1.75e-4, \end{array}$$

and the expected mean number of distinct neighbors at *D* < 3 was calculated as p.(N(1,7)+N(2,7)) ≈1.33. The value of p was used subsequently also in a binomial test to calculate the probabilities of finding equal or higher number of adjacent mimotopes (Table 1).

**Table 1:** Human public IgM repertoire (igome) selected mimotopes and their exact matches in the proteomes of SARS-CoV-2, SRAS-CoV, HCoV 229E and HCoV OC43.

	**Mimotopes**	**Protein_ID**	**Protein**	**Strain**	**Starting_pos**	**Number of Adjacent^3^ Mimotopes**
1	AQTGIAV	YP_009724389.1	orf1ab^1^ nsp5^2^	SARS-CoV-2	3,518	6^*^
2	TKGPHEF	YP_009724389.1	orf1ab nsp12	SARS-CoV-2	5,198	22^*^
3	TTLDSKT	YP_009724390.1	Spike	SARS-CoV-2	108	7^*^
4	HSYGIDL	NP_828849	orf1ab nsp2	SARS-CoV	134	5^*^
5	TTYNGYL	NP_828849	orf1ab nsp3	SARS-CoV	1,460	6^*^
6	AQTGIAV	NP_828849	orf1ab nsp5	SARS-CoV	3,495	6^*^
7	TKGPHEF	NP_828849	orf1ab nsp12	SARS-CoV	5,175	22^*^
8	VKGDDVR	NP_828851	Spike	SARS-CoV	389	8^*^
9	TTSTALG	NP_828851	Spike	SARS-CoV	922	6^*^
10	QLSLSMA	NP_828859	hypothetical	SARS-CoV	52	11^*^
11	GAGDAGH	NP_073549	orf1ab nsp3	229E	2,483	5^*^
12	QTSQALQ	NP_073551	Spike	229E	818	2
13	ANSFRLF	NP_073555	M protein	229E	96	2
14	NGSWVLN	AAR01012	orf1ab nsp3	OC43	2,987	2

1 *orf, open reading frame*;

2 *nsp, non-structural protein*.

3 *Adjacent mimotopes are considered those that have no more than 2 mismatches (Hamming distance <3)*,

* *p < 0.05, Binomial test, fdr adjusted*.

The structure of the spike of SARS-CoV-2 was recently published [6vsb.pdb (2)]. It was used to visualize the molecular context of the spike epitope found. The visualization of the structure and the calculation of the relative solvent exposed surface were done using UCSF Chimera, developed by the Resource for Biocomputing, Visualization, and Informatics at the University of California, San Francisco, with support from NIH P41-GM103311.

To demonstrate linear B cell epitope prediction uncertainty, we have reanalyzed data from He et al. (7) on patients' sera reactivity to SARS- CoV peptides comparing them to Bepipred (http://tools.iedb.org/bcell/help/#Bepipred2) scores of the same sequences.

## Results and Discussion

A simple comparison for exact matches to peptides from the SARS-CoV-2 proteome yielded 3 heptapeptides—two in the open reading frame 1ab (^3518^AQTGIAV^3524^ and ^5198^TKGPHEF^5204^) and one in the spike protein (^108^TTLDSKT^114^). The Expect value (E) is a parameter that describes the number of hits one can “expect” to see by chance when searching a database of a particular size. Essentially, the E value describes the random background noise (https://blast.ncbi.nlm.nih.gov/Blast.cgi?CMD=Web&PAGE_TYPE=BlastDocs&DOC_TYPE=FAQ#expect). The E value of search results with so short sequences is very high and the mere number of sequences is not statistically significant. Yet, this does not refute the fact that 3 heptapeptides which are operationally defined mimotopes of human preimmune antibodies, are part of the viral proteome and, thus, represent (parts of) possible epitopes. On the other hand, the mimotopes in the database sometimes form non-random clusters of homologous sequences much like the mimotopes selected by a single monoclonal antibody. Each one among 224,087 randomly selected heptamers should have on the average 1.33 homologous sequences in that same database that differ from it by up to 2 mismatches. As seen from Table 1, all SARS sequences but not those from trivial HCoV were members of clusters significantly greater than random (Binomial test, *p* < 0.05, false discovery rate adjusted). This is an indication that the presence of these sequences is non-random and they represent clusters of mimotopes representing well-represented individual (poly)specificities.

**Figure 1:**
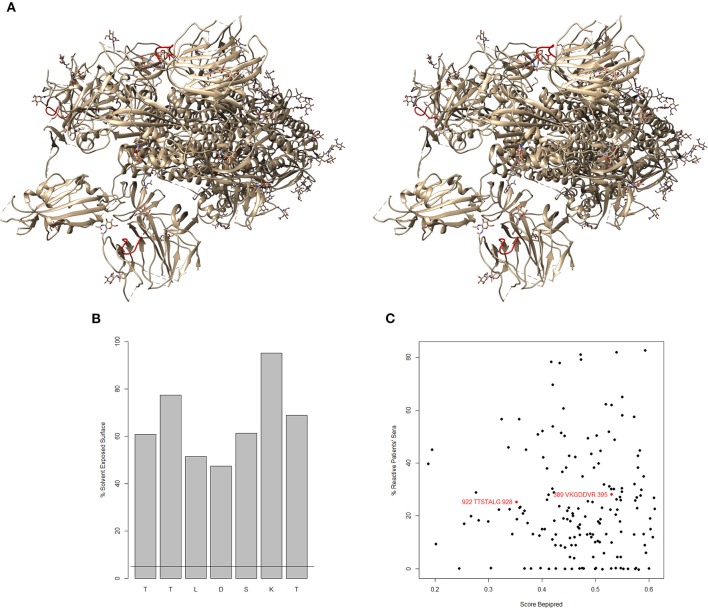
**(A)** Stereo view of the SARS-CoV-2 spike protein [6vsb.pdb (2)]. The putative natural IgM epitope ^108^TTLDSKT^114^ is colored red. **(B)** Relative solvent exposed surface by amino acid residue. The horizontal line marks the threshold of 5%. **(C)** Correlation of Bepipred score and the actual percentage of sera reactive with the same sequences from the spike of SARS-CoV [based on (7)]. The two predicted natural epitopes are overlaid in red. There is antibody reactivity in patients' sera to these epitopes although one of them has Bepipred score far below the threshold of 0.5.

An important prerequisite for the functionality of these epitopes is their degree of exposure to the solvent. The recently published structure of the spike (S protein) of SARS-CoV-2 (2) shows that ^108^TTLDSKT^114^ forms a loop exposed to the solvent (Figure 1A). The relative solvent exposed surface greatly exceeds the threshold of 5% for participating in contacts (Figure 1B). This loop is adjacent to the loop representing the epitope of the neutralizing antibody LCA60 on the SARS-CoV spike (8, 9). Presumably, it is similarly exposed further in the open conformation of the spike domains. The adjacent N-glycosylation sites are N165 and N234. Dependent on the size of the carbohydrate sidechains, they may partially occlude the epitope.

The closest sequences in the human proteome are ^540^tlTLDSKT^547^ of the prostate-specific transglutaminase (TGM4) and ^462^TTLDSKi^468^ of mucin-16 [also known as ovarian tumor marker CA125, Q8WXI7.3, (10)]. Both are on tumor associated antigens (10, 11). While TGM4 is an intracellular antigen, mucin-16 is highly accessible on cell surfaces and in a soluble form. The mucin sequence ^462^TTLDSKI^468^ is T/S biased, represents part of the highly O-glycosylated N-terminal part of mucin-16 and is predicted to be O-glycosylated itself. Normally, such mucin protein core epitopes are occluded by glycosylation and thus, cryptic with respect to immune tolerance. Yet, monoclonals to similar epitopes turned out to bind specifically to tumor expressed mucins (12–15) which are aberrantly/hypo glycosylated.

The sequences ^108^TTLDSKT^114^ has several exact matches in viruses outside the family Coronaviridae in hypothetical proteins of various phages. At least one of them infects *L. plantarum* which is a common species in the gut microbiome.

It is not surprising that the public IgM repertoire has clones potentially capable of binding to non-conserved regions of novel viruses. Similarly, the IgM igome contained sequences found also in SARS-CoV, although the epidemic was too restricted to be reflected in the antibody repertoires of the donors (Table 1). Furthermore, no signs of persistent antibody titers after exposure were observed. The representation of clones reactive with the trivial human coronaviruses 229E and OC43 was rather narrower than that of the unknown strains. Some of the epitopes were conserved between SARS-CoV and SARS-CoV-2 (AQTGIAV and TKGPHEF) but they were found in non-structural proteins and are hardly targets for neutralizing antibodies (Table 1). On the other hand, all potential epitopes found could play a role in targeting the viral proteins to specific B1 cells which produce the bulk of natural IgM. The latter are known to be excellent antigen presenting cells able to prime CD4^+^ T cells, and initiate Th1 immune responses (16–18) in antigen specific manner much like activated specific B2 cells (17). It has been shown that B1 cells secreted IgM is a non-redundant and essential arm of the humoral responses to influenza in mice (19). This implies that natural antibody epitopes might be essential components of subunit vaccines even though they may not represent typical dominant epitopes. The role of overlapping T and B cell epitopes is not clear except when the B cell receptor has a high enough affinity for the epitope to protect it during processing (20), but it is interesting that one of the SARS-CoV natural epitopes (^922^TTSTALG^928^) is also part of a CD4 T cell epitope in the context of HLA-DR B1^*^04:01 (21). Using the IEDB preferred method the epitope ^108^TTLDSKT^114^ is predicted to overlap a potential class II epitope in the context of HLA-DRB1^*^07:01, while two other potential epitopes just up- and downstream overlap it partially (in the context of HLA-DPA1^*^02:01/DPB1^*^01:01 and HLA-DRB1^*^04:01, HLA-DRB1^*^04:05 and HLA-DRB1^*^13:02, respectively). In this respect, maybe a more useful epitope would be the continuous sequence 99NIIRGWIFGTTLDSKTQSLLIVNNATNV126.

The current thinking separates the repertoire of natural and induced antibodies (22). The preimmune IgM mimotopes we describe could represent also epitopes of naïve B cell clones which may have undergone extrafollicular expansion poised to initiate also follicular immune responses. As to the capacity of these epitopes to induce fully mature antibody response, it is interesting to note that the two preimmune IgM epitopes found for the spike of SARS-CoV (922TTSTALG928 and 389VKGDDVR395) are proven antibody targets in approximately one fourth of the SARS patients (7). Thus, our mimotope library has the capacity to identify potential true precursor epitopes and not only natural antibody epitopes. Furthermore, a recent report indicates the importance of IgM antibodies in the control of the diseases in mild cases of COVID19 (23). Thus, it is quite possible that the SARS-CoV-2 spike epitope TTLDSKT is bound by B cells that will contribute to the induced immune response.

None of the *in silico* predicted epitopes (1–5) overlaps with ^108^TTLDSKT^114^ which is also specific to SARS-CoV-2. The correlation between the actual reactivities in SARS-CoV patients' sera and the Bepipred score (Figure 1C) confirms the low power of linear B cell epitope predicting algorithms, and underlies the necessity to base the proposals of new epitopes as much as possible on actual binding data.

These considerations make the novel SARS-CoV-2 epitopes valid targets in the search for a vaccine for COVID-19. The whole paradigm followed here focuses exclusively on the relatively rare linear epitopes. A lot more information about conformational epitopes may be hidden in the natural mimotope database but the approaches for sorting out clusters of mimotopes defining a conformational epitope are still being developed. The proposed actual preimmune IgM epitopes of SARS-CoV-2 can be instrumental both as parts of subunit vaccines or in the design of nanoparticle-based vaccines but also in the development of therapeutic monoclonal antibodies.

## Data Availability Statement

The datasets analyzed and the scripts for this study can be found in the GitHub Repository (https://github.com/ansts/SARS-CoV-2).

## Author Contributions

VS and AP: conceptualizing, manuscript preparation, and data analysis. PP: data analysis.

## Conflict of Interest

The authors declare that the research was conducted in the absence of any commercial or financial relationships that could be construed as a potential conflict of interest.

## References

[1] Zheng M Song L. Novel antibody epitopes dominate the antigenicity of spike glycoprotein in SARS-CoV-2 compared to SARS-CoV. Cell Mol Immunol. (2020). 10.1038/s41423-020-0385-z 32132669 PMC7091851

[2] Walls AC Park Y-J Tortorici MA Wall A McGuire AT Veesler D. Structure, function and antigenicity of the SARS-CoV-2 spike glycoprotein. Cell. (2020) 181:281–92.e6. 10.1016/j.cell.2020.02.058 32155444 PMC7102599

[3] Grifoni A Sidney J Zhang Y Scheuermann RH Peters B Sette A Candidate targets for immune responses to 2019-Novel Coronavirus (nCoV): sequence homology- and bioinformatic-based predictions. bioRxiv. (2020). 10.1101/2020.02.12.946087. PMC7142693 32183941

[4] Ahmed SF Quadeer AA McKay MR. Preliminary identification of potential vaccine targets for the COVID-19 coronavirus (SARS-CoV-2) based on SARS-CoV immunological studies. Viruses. (2020) 12:254. 10.3390/v12030254 32106567 PMC7150947

[5] Lucchese G. Epitopes for a 2019-nCoV vaccine. Cell Mol Immunol. (2020). 10.1038/s41423-020-0377-z 32094505 PMC7091830

[6] Pashov A Shivarov V Hadzhieva M Kostov V Ferdinandov D Heintz K-M. Diagnostic profiling of the human public IgM repertoire with scalable mimotope libraries. Front Immunol. (2019) 10:2796. 10.3389/fimmu.2019.02796 31849974 PMC6901697

[7] He Y Zhou Y Wu H Luo B Chen J Li W. Identification of immunodominant sites on the spike protein of severe acute respiratory syndrome (SARS) coronavirus: implication for developing SARS diagnostics and vaccines. J Immunol. (2004) 173:4050–7. 10.4049/jimmunol.173.6.4050 15356154

[8] Wrapp D Wang N Corbett KS Goldsmith JA Hsieh C-L Abiona O. Cryo-EM structure of the 2019-nCoV spike in the prefusion conformation. Science. (2020) 367:1260–3. 10.1126/science.abb2507 32075877 PMC7164637

[9] Walls AC Xiong X Park Y-J Tortorici MA Snijder J Quispe J. Unexpected receptor functional mimicry elucidates activation of coronavirus fusion. Cell. (2019) 176:1026–39.e15. 10.1016/j.cell.2018.12.028 30712865 PMC6751136

[10] Felder M Kapur A Gonzalez-Bosquet J Horibata S Heintz J Albrecht R. MUC16 (CA125): tumor biomarker to cancer therapy, a work in progress. Mol Cancer. (2014) 13:129. 10.1186/1476-4598-13-129 24886523 PMC4046138

[11] Cao Z Wang Y Liu Z-Y Zhang Z-S Ren S-C Yu Y-W. Overexpression of transglutaminase 4 and prostate cancer progression: a potential predictor of less favourable outcomes. Asian J Androl. (2013) 15:742–6. 10.1038/aja.2013.79 23974364 PMC3854051

[12] Burchell J Taylor-Papadimitriou J. Effect of modification of carbohydrate side chains on the reactivity of antibodies with core-protein epitopes of the MUC1 gene product. Epithelial Cell Biol. (1993) 2:155–62. 7505698

[13] Burchell J Gendler S Taylor-Papadimitriou J Girling A Lewis A Millis R. Development and characterization of breast cancer reactive monoclonal antibodies directed to the core protein of the human milk mucin. Cancer Res. (1987) 47:5476–82. 2443241

[14] Petrakou E Murray A Price MR. Epitope mapping of anti-MUC1 mucin protein core monoclonal antibodies. Tumour Biol. (1998) 19(Suppl. 1):21–9. 10.1159/000056501 9422085

[15] Zhou D Xu L Huang W Tonn T. Epitopes of MUC1 tandem repeats in cancer as revealed by antibody crystallography: toward glycopeptide signature-guided therapy. Molecules. (2018) 23:1326. 10.3390/molecules23061326 29857542 PMC6099590

[16] Baumgarth N. A two-phase model of B-cell activation. Immunol Rev. (2000) 176:171–80. 10.1034/j.1600-065x.2000.00606.x 11043776

[17] Popi AF Longo-Maugéri IM Mariano M. An overview of B-1 cells as antigen-presenting cells. Front Immunol. (2016) 7:138. 10.3389/fimmu.2016.00138 27148259 PMC4827000

[18] Hong S Zhang Z Liu H Tian M Zhu X Zhang Z. B cells are the dominant antigen-presenting cells that activate naive CD4+ T cells upon immunization with a virus-derived nanoparticle antigen. Immunity. (2018). 49:695–708.e4. 10.1016/j.immuni.2018.08.012 30291027

[19] Baumgarth N Herman OC Jager GC Brown LE Herzenberg LA Chen J. B-1 and B-2 cell-derived immunoglobulin M antibodies are nonredundant components of the protective response to influenza virus infection. J Exp Med. (2000) 192:271–80. 10.1084/jem.192.2.271 10899913 PMC2193249

[20] Simitsek PD Campbell DG Lanzavecchia A Fairweather N Watts C. Modulation of antigen processing by bound antibodies can boost or suppress class II major histocompatibility complex presentation of different T cell determinants. J Exp Med. (1995) 181:1957–63. 7539034 10.1084/jem.181.6.1957 PMC2192058

[21] Yang J James E Roti M Huston L Gebe JA Kwok WW. Searching immunodominant epitopes prior to epidemic: HLA class II-restricted SARS-CoV spike protein epitopes in unexposed individuals. Int Immunol. (2009) 21:63–71. 10.1093/intimm/dxn124 19050106 PMC2638843

[22] Baumgarth N Tung JW Herzenberg LA. Inherent specificities in natural antibodies: a key to immune defense against pathogen invasion. Springer Semin Immunopathol. (2005) 26:347–62. 10.1007/s00281-004-0182-2 15633017

[23] Thevarajan I Nguyen THO Koutsakos M Druce J Caly L van de Sandt CE. Breadth of concomitant immune responses prior to patient recovery: a case report of non-severe COVID-19. Nat Med. (2020) 26:453–5. 10.1038/s41591-020-0819-2 32284614 PMC7095036

